# Synergistic effects of diazotrophs and arbuscular mycorrhizal fungi on soil biological nitrogen fixation after three decades of fertilization

**DOI:** 10.1002/imt2.81

**Published:** 2023-01-27

**Authors:** Guopeng Zhou, Kunkun Fan, Guilong Li, Songjuan Gao, Danna Chang, Ting Liang, Shun Li, Hai Liang, Jiudong Zhang, Zongxian Che, Weidong Cao

**Affiliations:** ^1^ Key Laboratory of Plant Nutrition and Fertilizer, Ministry of Agriculture and Rural Affairs/Institute of Agricultural Resources and Regional Planning Chinese Academy of Agricultural Sciences Beijing China; ^2^ State Key Laboratory of Soil and Sustainable Agriculture, Institute of Soil Science Chinese Academy of Sciences Nanjing China; ^3^ Institute of Soil & Fertilizer and Resource & Environment Jiangxi Academy of Agricultural Sciences Nanchang China; ^4^ College of Resources and Environmental Sciences Nanjing Agricultural University Nanjing China; ^5^ Institute of Soil and Fertilizer and Water‐saving Agriculture Gansu Academy of Agriculture Science Lanzhou China

**Keywords:** farmland system, interspecies relationship, long‐term fertilization, key ecological cluster

## Abstract

Biological nitrogen (N) fixation (BNF) via diazotrophs is an important ecological process for the conversion of atmospheric N to biologically available N. Although soil diazotrophs play a dominant role in BNF and arbuscular mycorrhizal fungi (AMF) serve as helpers to favor BNF, the response of soil BNF and diazotrophic communities to different long‐term fertilizations and the role of AMF in diazotrophs‐driven BNF are poorly understood. Herein, a 33‐year fertilization experiment in a wheat–maize intercropping system was conducted to investigate the changes in soil BNF rates, diazotrophic and AMF communities, and their interactions after long‐term representative fertilization (chemical fertilizer, cow manure, wheat straw, and green manure). We found a remarkable increase in soil BNF rates after more than three decades of fertilization compared with nonfertilized soil, and the green manure treatment rendered the highest enhancement. The functionality strengthening was mainly associated with the increase in the absolute abundance of diazotrophs and AMF and the relative abundance of the key ecological cluster of Module #0 (gained from the co‐occurrence network of diazotrophic and AMF species) with dominant diazotrophs such as *Skermanella* and *Azospirillum*. Furthermore, although the positive correlations between diazotrophs and AMF were reduced under long‐term organic fertilization regimes, green manuring could reverse the decline within Module #0, and this had a positive relationship with the BNF rate. This study suggests that long‐term fertilization could promote N fixation and select specific groups of N fixers and their helpers in certain areas. Our work provides solid evidence that N fixation and certain groups of diazotrophic and AMF taxa and their interspecies relationship will be largely favored after the fertilized strategy of green manure.

## Highlights

- Long‐term fertilization enhanced soil biological nitrogen fixation (BNF).
- Diazotrophic and arbuscular mycorrhizal fungal (AMF) abundance is positively linked to BNF.
- Key ecological cluster consisting of diazotrophs and AMF played vital roles in soil BNF.
- Long‐term fertilization reduced the synergistic functions of diazotrophs and AMF.
- Green manure strengthened the synergistic function within the key ecological cluster.

## Graphical abstract

In this study, a 33‐year fertilization experiment was conducted to investigate the changes in soil BNF rates, diazotrophic and AMF communities, and their interactions after various long‐term fertilizations. We found a remarkable increase in soil BNF rates after more than three decades of fertilization compared with nonfertilized soil, and the green manure treatment rendered the highest enhancement.

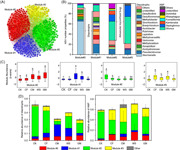

## INTRODUCTION

Biological nitrogen (N) fixation (BNF), a vital ecological process for the conversion of atmospheric N to biologically available N, occurs via symbiotic or asymbiotic (free‐living) pathways and is responsible for fixing 40–100 Tg N year^−1^ to the terrestrial ecosystem [1]. Although symbiotic BNF contributes a major proportion of total BNF [2], asymbiotic BNF occurs nearly ubiquitously in the soil, litter layer, and even stalk and leaves of plants and is equally crucial for the terrestrial N budgets of the earth [2–4]; for instance, farmland soil can contribute up to 22–53 kg N ha^−1^ year^−1^ [5]. Nevertheless, large amounts of inorganic or organic N inputs challenge the contributions of BNF and the associated microorganisms, as N fixers (diazotrophs) theoretically downregulate BNF rates or are outcompeted by nondiazotrophs under N‐rich conditions [6, 7], owing to the fact that N uptake directly from the soil is less energetically expensive than BNF [8]. Unfortunately, there is no uniform evidence of the inhibitory effect of fertilizers on soil asymbiotic BNF in agricultural ecosystems [9–11], that is, increased N availability having stimulatory [10] or suppressive [9] effects on soil BNF. A study based on forest systems also reported that individual nutrient availability (including N or phosphorous) was not the best predictor of soil BNF [12]. Although the effects of environmental factors on BNF are well‐documented [2, 9, 12, 13], our knowledge regarding the factors that control diazotrophs on soil BNF remains very poor, especially in the agricultural system under dramatic human disturbances.

Although diazotrophs theoretically have a strong advantage in a nutrient‐deficient environment, the effect of fertilization on the diazotrophic community remains inconclusive, that is, the changes in richness and diversity of diazotrophs after organic or inorganic fertilization varied in previous fertilization experiments [14–17]. Soil diazotrophs are also well known for the possible existence of different diazotrophic taxa owing to habitat variation [18, 19]. For instance, short‐term N fertilization can increase the abundance of fast‐growing (copiotrophic) diazotrophs such as *Sphingomonas* and *Burkholderia*, classified as Alphaproteobacteria and Betaproteobacteria [20, 21]. Whereas long‐term fertilizations, including N fertilizing, increased the relative abundance of *Geobacter* [10], belonging to Deltaproteobacteria, which is often categorized as oligotrophic bacteria [22]. According to these findings, predicting the changes in the diazotrophic community by referring to their nutritional features or living habits might be not reasonable. Therefore, it is important to have more knowledge about the changes in diazotrophic communities due to fertilizations for the development of management frameworks to provide fertilization strategies for controlling diazotrophic taxa involved in the high efficiency of soil BNF.

Arbuscular mycorrhizal fungi (AMF), belonging to the phylum Glomeromycota, are well known to be of numerous advantages to their host plants, such as improving nutrients uptake, increasing plant tolerance, and stimulating carbon (C) and mineral nutrient cycles [23, 24]. AMF can also play an important role in modifying BNF by influencing diazotrophs in the plant rhizosphere and hyphosphere [25, 26]. For instance, AMF can mainly produce hyphae to offer highly efficient corridors for available nutrients, thus favoring nutrient communications with diazotrophs [27] or promoting the growth of heterotrophic nondiazotrophs to modify the community of diazotrophs [28, 29]. Evidentially, AMF facilitates N fixation of diazotrophs such as *Azospirillum* and *Frankia* [30]. Recent studies also reported that synergies rather than antagonisms between AMF and diazotrophs prevail in the interspecies relationship [31], but their relationships were diminished in high N‐treated soils compared to those in low N‐treated soils [16]. N‐rich environment suppresses diazotrophs and AMF growth in soil [32, 33], which might be the most important reason for the diminishment of the interspecies relationship. However, although previous study has reported that fertilization would regulate the interspecies relationship between diazotrophs and AMF [16], the effect of the changes on soil BNF is unclear. Thus, improving the current knowledge of the role of AMF and diazotrophs interactions in regulating soil BNF is essential for probing this aspect.

Recently, studies have utilized microbial co‐occurrence networks to explore the interactions among taxa [34]. It has been reported that in the soil ecosystem, complex microbial co‐occurrence networks could be divided into certain ecological clusters (modules) to maintain community composition and functions [33, 35, 36]. For example, Wang et al. showed that soil multifunctionality was driven by biodiversity and community dynamics in ecological clusters of soil organisms, rather than the overall communities [36], and Fan et al. demonstrated that soil BNF was associated with the relative abundance of keystone and phylogenetically clustered N fixers [9]. Therefore, an ecological cluster based on microbial co‐occurrence network analysis would be a major tool to infer the potential relationship between soil microbiome and ecosystem functions. However, to date, few studies have employed the tool to investigate the ecological relationship between diazotrophs and arbuscular mycorrhizal fungi and their interactions in modifying soil BNF. Similar studies will advance our knowledge of the synergic effects of diazotrophs and AMF on soil BNF.

Thus, in this study, we used a long‐term field experiment setup in 1988, which included representative fertilizations by chemical fertilizer, cow manure, wheat straw, and green manure, and employed the Illumina MiSeq high‐throughput sequencing technology to examine the linkage between soil BNF rates and diazotrophs and their helpers (AMF). We hypothesized that (H1) long‐term fertilization would inhibit the soil BNF and drastically alter the diazotrophic community structure that may no longer be required to fix atmospheric N, (H2) compared to the entire microbial community, unique co‐occurring ecological clusters of diazotrophs and AMF might predict BNF rates better, and (H3) long‐term fertilization would lower the synergistic (positive) relationship between diazotrophs and AMF owing to the nutrient‐rich environment in the fertilization regime.

## RESULTS

### Biological nitrogen fixation, diazotrophic, and arbuscular mycorrhizal fungi communities under long‐term fertilization regimes

**Figure 1:**
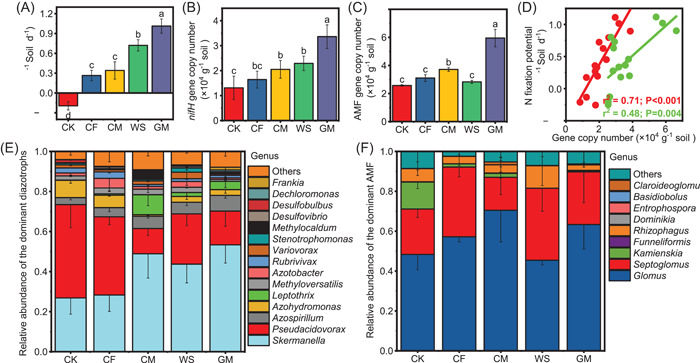
Soil biological nitrogen fixation potential and its related microbes after almost three decades of fertilization. (A) Biological nitrogen fixation (BNF) rates, (B) diazotrophic absolute abundance (based on the *nifH* gene copy number), and (C) arbuscular mycorrhizal fungal absolute abundance (based on the AMF gene copy number) after 33 years of fertilization. (D) The linear relationships between BNF rates and diazotrophs and AMF absolute abundance. The regressions of diazotrophs and AMF are represented by red and green plots, respectively. (E and F) The relative abundances of the dominant diazotrophs and AMF at the genus level under different fertilization regimes. Different lowercase letters denote significant differences at *p* < 0.05 (LSD test) between treatments. AMF, arbuscular mycorrhizal fungi; CF, chemical fertilizer; CK, nonfertilization; CM, cow manure; GM, green manure; WS, wheat straw.

The BNF rates were significantly (*p* < 0.05) promoted relative to the CK after more than three decades of fertilization, showing an order of GM > WS > CM ≈ CF > CK (Figure 1A). Long‐term organic fertilization (including CM, WS, and GM) could significantly (*p* < 0.05) increase the absolute abundance of diazotrophs and AMF (apart from WS treatment for AMF) (Figure 1B,C). The diazotrophic and AMF absolute abundance presented strong and positive correlations with BNF rates (*r* ^2^ = 0.71, *p* < 0.001; *r* ^2^ = 0.48, *p* = 0.004, respectively, Figure 1D).

Permutational multivariate analysis of variance (PERMANOVA) indicated that various long‐term fertilizations remarkably shaped community structures of diazotrophs and AMF (*R* = 0.52, *p* = 0.001; *R* = 0.32, *p* = 0.001, respectively, Supporting Information: Table S3). For diazotrophs, long‐term GM significantly (*p* < 0.05) increased the relative abundance of *Skermanella* and *Azospirillum*, whereas organic fertilization significantly (*p* < 0.05) decreased the relative abundance of *Pseudacidovorax* (apart from WS), *Azohydromonas*, and *Desulfobulbus* compared with CK (Figure 1E, and Supporting Information: Figure S4). Among AMF, all the fertilization treatments markedly (*p* < 0.05) decreased the relative abundance of *Kamienskia*; meanwhile, *Septoglomus* was highly enriched in the CF treatment (Figure 1F, and Supporting Information: Figure S4). Organic fertilization significantly (*p* < 0.05) increased the observed species and Chao1 indexes of diazotrophs, while all fertilization treatments (apart from GM) significantly (*p* < 0.01) decreased both indexes of AMF (and Supporting Information: Table S4). Mantel test showed that soil pH, total nutrients (including soil organic carbon [SOC], total nitrogen [TN], and Total phosphorous [TP]), available nutrients (including mineral N and AP), and their ratio significantly (*p* < 0.05) shaped the diazotrophic community; however, the AMF community was mainly modulated by soil TP and available N‐to‐P ratios, such as nitrite N‐to‐AP ratio and mineral N‐to‐AP ratio (Supporting Information: Figure S5A).

### Co‐occurrence pattern of diazotrophs and arbuscular mycorrhizal fungi under long‐term fertilization regimes

**Figure 2:**
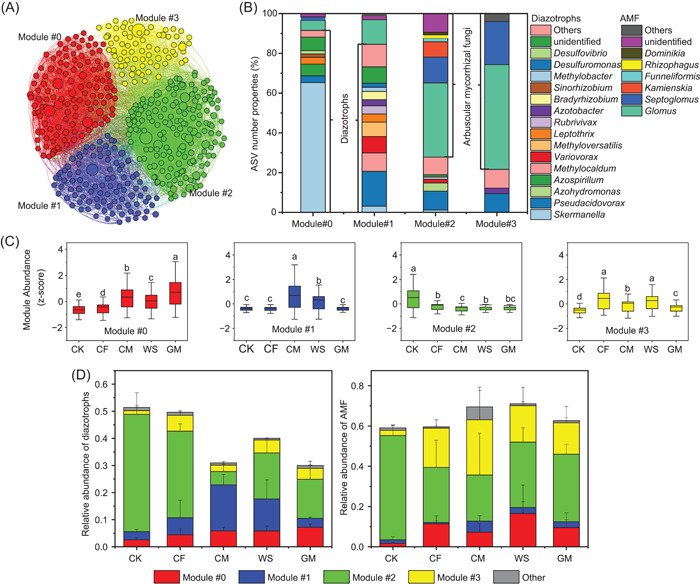
Ecological clusters based on co‐occurrence network interactions of diazotrophs and AMF. (A) Correlation network including multiple ecological clusters (Modules #0 to #3). (B) ASV number properties of the dominant diazotrophic and AMF genera in the multiple ecological clusters. (C) Relative abundance of the ecological clusters under different fertilization regimes. (D) Relative abundance of ASVs with positive relationships between diazotrophs and AMF in the co‐occurrence network. Different lowercase letters denote significant differences at *p* < 0.05 (LSD test) between treatments. AMF, arbuscular mycorrhizal fungi; ASV, amplicon sequence variant; CF, chemical fertilizer; CK, nonfertilization; CM, cow manure; GM, green manure; WS, wheat straw.

A co‐occurrence network was built based on the relative abundance of diazotrophic and AMF ASVs as nodes and the correlation between nodes based on Spearman's rank correlation coefficient (Supporting Information: Figure S6A). According to the network topological parameters, the numbers of nodes and edges were lower in CF than in other treatments, suggesting that long‐term CF treatment decreased the complexity of the soil microbial community network (Supporting Information: Figure S6B). Then, four ecological clusters (including Modules #0, #1, #2, and #3) of strongly co‐occurring diazotrophs and AMF were further identified from the co‐occurrence network (Figure 2A, Supporting Information: Table S5). Each module was constituted of multiple diazotrophic and AMF species belonging to different genera (Figure 2B). Taken as a whole, diazotrophs dominated Modules #0 and #1, AMF dominated Modules #2 and #3; Modules #0 and #3 had lower diversities of microbial genera than Modules #1 and #2 (Figure 2B). Specifically, *Skermanella* and *Azospirillum* were the most dominant diazotrophic genera in Module #0, and diazotrophic *Pseudacidovorax*, *Methylocaldum*, *Variovorax*, and *Methyloversatilis* dominated Module #1. Modules #2 and #3 were dominated by AMF genera *Glomus* and *Septoglomus*, and in all four modules, *Glomus* was the most abundant genus of AMF (Figure 2B). Long‐term fertilization significantly (*p* < 0.05) increased the relative abundances of Modules #0 and #3 and reduced that of Module #2, with the greatest enhancement in Module #0 due to the GM treatment (Figure 2C). Similar relative abundances were observed for separate diazotrophs and AMF within each ecological cluster (Supporting Information: Figure S7).

**Figure 3:**
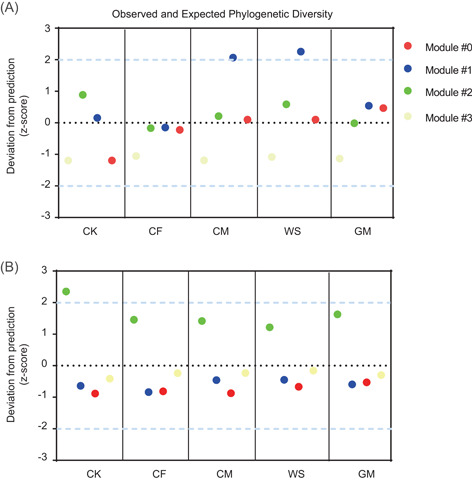
The standardized difference between observed and expected phylogenetic diversity. Panels (A) and (B) are for diazotrophs and AMF, respectively. The dotted black line indicates the expected PD for each treatment, and the dashed gray lines indicate the 95% confidence interval. AMF, arbuscular mycorrhizal fungi; CF, chemical fertilizer; CK, nonfertilization; CM, cow manure; GM, green manure; PD, phylogenetic diversity; WS, wheat straw.

We analyzed the PD of diazotrophic and AMF ASVs within each module and then compared the observed PD with the expected PD for each module (Figure 3). The observed PD for diazotrophs in Modules #0, #2, and #3, and AMF in Modules #0, #1, and #3 followed the random predictions (>−2 and <2) across all treatments. However, the observed PD for diazotrophs in Module #1 under long‐term CM and WS regimes and AMF in Module #2 under the nonfertilization regime deviated significantly (*p* < 0.05) above the random prediction (>2). These findings indicate that long‐term CM and WS may have been selected against the diazotrophs associated with Module #1, while long‐term fertilization was selected against the AMF associated with Module #2.

Assessment of the correlations among different ecological clusters and within each ecological cluster revealed a strongly synergistic (positive) relationship between Modules #0 and #1, while Module #2 presented an antagonistic (negative) correlation with other modules (including Modules #0, #1, and #3) (Supporting Information: Table S5). Evaluation of the role of AMF in BNF by closely analyzing the positive relationship between diazotrophic and AMF species revealed that the co‐occurrence network had 137 AMF nodes (dominated by *Glomus* sp.) which were positively linked to 152 diazotrophic nodes (dominated by *Pseudacidovorax* sp. and *Skermanella* sp.) (Supporting Information: Table S6 and S7), and most positive links existed in Modules #1 and #2. Long‐term organic fertilization reduced the relative abundance of diazotrophs which were positively linked to AMF—particularly under CM and GM additions (Figure 2D, Supporting Information: Table S8). Meanwhile, these diazotrophic communities (positively linked to AMF) were regulated by soil nutrients, and the relative abundances were negatively correlated with SOC, TN, TP, AP, and so on (Supporting Information: Figure S8A and S9). These results suggest that high soil fertility would reduce the relative abundance of diazotrophs (positively linked to AMF), especially those in Module #2 (Figure 2D). Interestingly, long‐term GM treatment significantly (*p* < 0.05) enhanced the relative abundance of diazotrophs (positively linked to AMF) in Module #0 by decreasing the soil mineral N‐to‐AP ratio (Figure 2D, and Supporting Information: Figure S8B and S9).

### Linking key ecological clusters to biological nitrogen fixation under long‐term fertilization regimes

**Figure 4:**
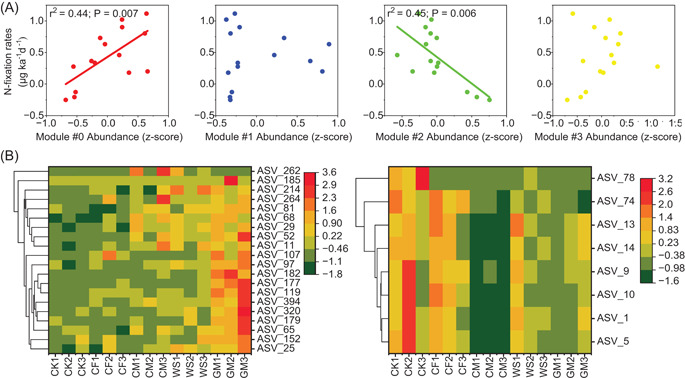
Relationship between the soil biological nitrogen fixation potential and ecological cluster. (A) The linear relationships between the BNF rates and the relative abundance of the key ecological clusters. (B) Amplicon sequence variant abundance properties of significant diazotrophic phylotypes for BNF rates based on the Random Forest model in the key ecological clusters (Modules #0 and #3). The left and right plots represent Modules #0 and #3, respectively. BNF, biological nitrogen fixation; CF, chemical fertilizer; CK, nonfertilization; CM, cow manure; GM, green manure; WS, wheat straw.

Among these modules, the relative abundance of Module #0 was significantly (*r* ^2^ = 0.44, *p* = 0.007) and positively correlated with BNF rates, while that of Module #2 was strongly (*r* ^2^ = 0.45, *p* = 0.006) and negatively correlated with BNF rates (Figure 4A). Given the contribution of Modules #0 and #2 to BNF rate, both modules were referred to as key ecological clusters (keystone phylotypes) hereafter. Assessment of the N fixers, which were highly and positively linked to BNF rates via Random Forest regression in each key ecological cluster, revealed that 20 and 8 of diazotrophic phylotypes in Modules #0 and #2 were enriched in organic fertilization (particularly in GM) and control (CK), respectively (Figure 4B and Supporting Information: Table S9). In Modules #0 and #2, 15% (3/20) and 75% (6/8) of diazotrophic phylotypes, respectively, showed a positive association with AMF (Supporting Information: Table S9), thus confirming the hypothesis that AMF is an important helper for diazotrophs to fix N_2_ in soil with low fertility.

**Figure 5:**
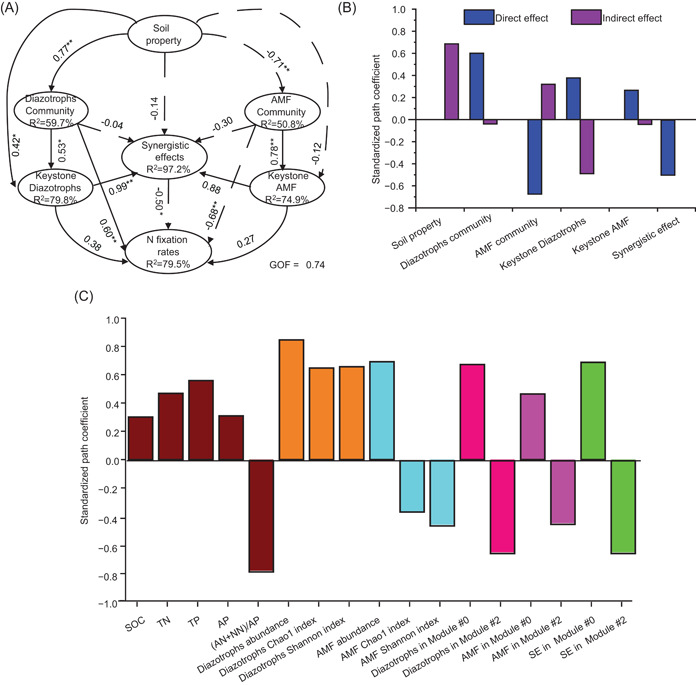
Partial least squares path model describing the biotic and abiotic factors affecting the biological nitrogen fixation potential. (A) A partial least squares path model describing the effects of soil property, diazotrophs, and AMF on the biological nitrogen fixation rates. Latent variable of soil property includes SOC, TN, TP, AP contents, and the mineral N (ammonium and nitrate nitrogen, AN + NN)‐to‐AP ratio. Latent variable of diazotrophs and AMF community consist of corresponding microbial abundance, Observed species and Shannon indexes, respectively. Latent variable of the synergistic effect is composed of the relative abundance of diazotrophs which are positively linked to AMF in the key ecological cluster. Path coefficients are labeled beside the arrow lines; solid and dashed arrows indicate positive and negative effects, respectively (*p < 0.05 and **p < 0.01). (B) The standardized path coefficients for direct and indirect effects of treatments, soil properties, diazotrophs, and AMF on nitrogen fixation rates. (C) The path model cross‐loading effect of each variable on soil biological nitrogen fixation. AMF, arbuscular mycorrhizal fungi; AP, available phosphorus; SE, synergistic effects; SOC, soil organic carbon; TN, total nitrogen; TP, total phosphorus.

The PLS‐PM analysis was then employed to further examine the associations between key ecological clusters and soil BNF rates and acquire a more exhaustive understanding of direct and indirect effects of soil property, communities of diazotrophs and AMF, and their synergistic function when simultaneously considering the multiple factors. The diazotrophic community (including absolute abundance and alpha diversity) had direct positive effects on BNF rates, while a significant (*p* < 0.05) negative association was found between the alpha diversity (including Observed species and Shannon indexes) of AMF and BNF rates (Figure 5A,B). Notably, no significant association was observed between the latent variables of keystone phylotypes and BNF rates because latent variables consisted of two group phylotypes (Modules #0 and #2) with opposite functions. However, the cross‐loading effects suggested that the whole diazotrophs and diazotrophs positively linked to AMF in key ecological clusters had a strong effect on BNF rates (Figure 5C), which also were confirmed by Spearman's correlation analysis (Supporting Information: Figure S9). From a managerial point of view, the positive effects of long‐term fertilization on diazotrophs in Module #0 seemed to be strengthened when the GM strategy was adopted.

## DISCUSSION

Contrary to our hypothesis that BNF is inhibited by long‐term fertilization (H1), we found that BNF rates increased in all fertilization treatments and peaked in GM treatment, where the soil TN and mineral N contents were higher than those in control. Recent studies have also reported similar results at a fine spatial and temporal scale, for example, little evidence of N availability controls on soil BNF [10, 37]. These results are extensions of a few previous findings that organic fertilization and P addition increased soil BNF rates [10, 38–40] and contrast with many previous findings that BNF was suppressed with the increase in soil TN or mineral N content caused by N‐fertilization, deposition, and so on [9, 13, 41]. The reasons for the increase in BNF rates in our study site after inorganic or organic fertilization (although it increased TN or mineral N content) may be related to a low background SOC content and P limitation. In the present study, the diazotrophic communities were dominantly heterotrophic and relied on soil C to fuel BNF [2, 42], which resulted in the greater potential effects of the increase in SOC content on diazotrophs than those of N enrichment [12]. Meanwhile, P can regulate both the substantial ATP demand of N fixation and the need to regulate O_2_ near the O_2_‐sensitive nitrogenase enzyme responsible for catalyzing N fixation [43], which may be responsible for increased N fixation. Therefore, both organic C and P limitations seemed to be crucial factors that resulted in an increase of N fixation in fertilization treatments in this study. Additionally, long‐term fertilization also changed the soil C:N and N:P ratios, thus resulting in great variations in BNF rates. Our results are not fully congruent with prior theories [12] and observations [10, 13, 44] that the BNF rate increases with the increase or reduction of N:P or C:(N:P) ratios, such as in CM treatment with the lowest and highest N:P or C:(N:P) ratios but with a lower BNF rate than WS and GM. Overall, our results suggest that at a specific and small area of fertilization experiment, N availability or stoichiometry theory alone are not good indicators of BNF as suggested by larger scale studies [2, 12]. We further focused on the community of N fixer and its helper to reveal the underlying mechanisms of variations in BNF.

Since many of the microorganisms participating in BNF are heterotrophic or mixotrophic, the addition of external organic matter provides a source of energy and nutrients to support growth [45]. Our study also provides robust evidence that, after 33 years of organic fertilization, there was a remarkable increase in the diazotrophic absolute abundance (25% to 156% increase), and it was positively associated with BNF rates. The PLS‐PM also provided further evidence that diazotrophic absolute abundance was the first, most robust effect on BNF rates. Interestingly, instead of high available P content from fertilizer reducing the absolute abundance of AMF [46], long‐term fertilization, especially the CM and GM treatments, actually enriched the AMF community. One important reason is that AMFs are obligate biotrophic fungi and can proliferate extensively in nutrient‐rich organic materials [47, 48]. In addition, long‐term GM resulted in the highest absolute abundance of AMF (as a helper of N fixation) which could form hyphae to provide highly efficient transport corridors for chemical compounds (especially P) to support the BNF by diazotrophs [27, 31]. These results agree with the previous findings of Li et al. [49] and Guo et al. [50] that green manure or legume in agriculture system has the advantage of enriching diazotrophs and AMF, which indicate that the potential effects of biotic factors (such as GM root exudates) on the microbial community may be larger than the variations in the soil environment.

To further analyze the diazotrophic and AMF communities, we constructed their co‐occurrence network and found four main ecological clusters (modules); then, we calculated the PD of each module. Our results showed that long‐term CM and WS modified the diazotrophic communities within Module #1 from phylogenetic randomness to phylogenetic over‐dispersion [51], suggesting that these diazotrophic phylotypes were largely distantly related and had a great competitive exclusion in both fertilization regimes [52]. The shift in observed PD of Module #1 was tightly linked with a large emergence of copiotrophic genera such as *Methylocaldum*, *Methyloversatilis*, and *Leptothrix* under CM and WS regimes, most of which belong to Betaproteobacteria (often classified as copiotrophic phylotypes [20]). These results suggest that a large amount of organic matter input would result in diazotrophs with similar features and competition for the same niches or resources. However, we observed that long‐term fertilization resulted in a shift from phylogenetic over‐dispersion to randomness for the AMF within Module #2 [51]. The changes in phylogenetic structure in Module #2 were closely linked with a large loss in *Kamienskia* sp., *Septoglomus* sp., and *Glomus* sp. Whether these species in our study site were strong competitors for resources remained to be determined through further investigation; nonetheless, this finding elucidated that long‐term fertilization changes the dominant ecological processes responsible for the assembly of AMF communities toward increased coexistence with the enrichment of resources. We consider that this phenomenon of interspecies competition (limited resources) under certain fertilization regimes would be partly regarded as a reason for a lower soil BNF rate than other treatments.

Our PLS‐PM suggested that long‐term fertilization indirectly increased the BNF rate by increasing and decreasing the relative abundance, respectively, of key ecological clusters of Modules #0 and #2. This finding indicates that long‐term fertilization directionally selects certain co‐occurring ecological clusters of diazotrophs and AMF rather than the entire microbial community to promote soil BNF, which supported our second hypothesis (H2). In Module #0, diazotrophic phylotypes within the dominant genera *Skermanella* and *Azospirillum* are known to be facultative fixers [53, 54], which could utilize nutrients from the fertilized soil to facilitate vegetative growth. These genera belong to the class Alphaproteobacteria, which are often classified as copiotrophic populations [55], and they can be modified to have a faster growth rate when substrates are sufficient [56]. Our results implied that the copiotrophic diazotrophs within Module #0 still maintained a high ability of BNF, which dispelled our previous knowledge that environmental resource richness would reduce the BNF ability of copiotrophic fixers [9, 57]. This phenomenon could be explained by the fact that fertilization results in rich soil resources that often provide a large habitable niche (e.g., a higher standing stock of soils) and low O_2_ environment (e.g., by constructing O_2_‐poor aggregate structure and by increasing respiration rates to locally deplete O_2_) for diazotrophs, both of which theoretically favor soil BNF [2, 43, 58].

Unlike Module #0, the diazotrophs within Module #2 were the losers under fertilization regimes. Moreover, long‐term fertilization increased soil BNF rates by decreasing the relative abundance of diazotrophs within Module #2, suggesting that diazotrophic phylotypes within Module #2 may have a low BNF capacity. In this connection, we then recognized a list of loser phylotypes, including *Pseudacidovorax* and *Azohydromonas*, whose relative abundance was high under a long‐term nonfertilization regime. This finding has a couple of possible explanations. Although genera *Pseudacidovorax* and *Azohydromonas* belong to the class Betaproteobacteria, both presented oligotrophic features. Likewise, previous studies have reported that these taxa are often enriched in nutrient‐poor environments [59, 60]. In addition, diazotrophic phylotypes within Module #2 could have a lower capacity to upregulate the BNF rate, as they are limited under low P and C conditions. This idea is supported by the more positive relationships between diazotrophs and AMF within the Module #2 ecological cluster.

Increasing evidence suggests that AMF can serve as helpers to favor BNF by transporting nutrients and promoting nutrient interaction with the diazotrophic community [25–28]. One recent study reported the existence of synergies rather than antagonisms between root‐associated diazotrophs and AMF in the interspecies relations in the rhizosphere in a forest ecosystem [31], which were also supported by our results. Although the positive associations between diazotrophs and AMF have been confirmed [31], our findings further extend the application of this knowledge to bulk soil. Interestingly, the relative abundance of diazotrophs cooperating with AMF appeared negatively associated with long‐term fertilizations, suggesting that this cooperation pattern could be reduced in a nutrient‐rich environment. This supported our third hypothesis that long‐term fertilizations would lower the synergistic relationships between diazotrophs and AMF (H3). More direct pieces of evidence were found in the key ecological clusters (Modules #0 and #2), where the diazotrophs and AMF within Module #0 enriched in organic fertilization treatments had fewer positive links than those within Module #2 enriched in nonfertilization treatments. However, long‐term GM significantly improved the cooperation between diazotrophs and AMF in Module #0, which could be regarded as another important reason for favoring soil BNF. Supporting this idea, the relative abundance of diazotrophs linking to AMF was significantly and positively correlated with BNF rates.

Although this study revealed that the relative abundance of key ecological clusters played an important role in maintaining soil BNF, it was not verified by direct evidence (e.g., gained from inoculation experiment of key phylotypes). In addition, the synergistic effects of diazotrophs and arbuscular mycorrhizal fungi that are based only on positive correlations could yield spurious results and cannot be automatically interpreted as accurate proof. Consequently, it may not be possible to comprehensively depict the microbial interactions under real‐world conditions [61]. However, the above results based on correlation are still essential for estimating potential species interrelationships or their functionality within complex environments and, in turn, for providing new clues for future studies on microbes‐driven soil BNF.

## CONCLUSION

This study provides robust evidence that long‐term fertilization dramatically promoted soil biological nitrogen fixation (BNF) rates, essentially supported by the absolute abundance of diazotrophs and AMF and the relative abundance of their key ecological cluster (Module #0, gained from co‐occurrence network of diazotrophic and AMF species), ultimately challenging the first hypothesis (H1) that long‐term fertilization suppresses BNF. In particular, the relative abundance of Module #0 could contribute to soil BNF by mainly enriching the diazotrophic *Skermanella* and *Azospirillum*, providing strong support for the second hypothesis (H2) that the effects of diazotrophic community on BNF are due to the important role of key phylotypes within a community. Additionally, the synergistic relationships between diazotrophs and AMF were reduced under long‐term organic fertilization regimes, which is corresponding to the third hypothesis (H3) that nutrient‐rich conditions reduce the positive associations between diazotrophs and AMF; however, green manure could reverse this trend within Module #0, favoring soil BNF. These findings extend our knowledge about the linkage between BNF and diazotrophs as well as their helper (AMF) and highlight the importance of relative abundance of the key ecological cluster of diazotrophs and AMF in maintaining soil BNF, suggesting the possibility of improving soil BNF by mediating soil microbial keystone phylotypes in intensified agricultural ecosystems. Further research isolating keystone phylotypes, designing simple microbiomes, as well as inoculating them in soil, is needed to verify the ecological functions of the key ecological cluster.

## METHODS

### Experimental design and sample collection

For the experimental setup, wheat–maize intercropping was conducted in 1988 in Wuwei County (38°37′N, 102°40′E, 1504 m elevation), Gansu Province, China. The soil type is irrigated desert soil, with 36.9% sand, 55.4% silt, and 7.7% clay, has an original pH (H_2_O) of 8.8 [62], and is classified as Inceptisols according to the Soil Survey Staff [63]. This region has a typical temperate continental climate, with annual potential evaporation of 2021 mm, an average temperature of 7.7°C, and precipitation of 150 mm for recent decades. The meteorological data of the growing season (from March to October) in 2020 was described in Supporting Information: Figure S1. Five types of treatments were conducted with three replicates in a completely randomized block design (each plot was of size 6.9 × 4.5 m^2^), including the following: (1) CK: control (nonfertilization), (2) CF: plots with urea (375 kg N ha^−1^ a^−1^), (3) CM: plots with cow manure (120000 kg ha^−1^ a^−1^), (4) WS: plots with wheat straw (10500 kg ha^−1^ a^−1^), and (5) GM: plots with fresh hairy vetch (*Vicia villosa* Roth L.) as green manure (45000 kg ha^−1^ a^−1^). To each treatment plot, superphosphate (150 kg P_2_O_5_ ha^−1^ a^−1^) was added except for the CK. In CM and WS, cow manure and wheat straw, respectively, were applied in late March annually and mixed thoroughly with topsoil. For the GM treatment, hairy vetch was annually sown with 120 kg ha^−1^ seed rate after spring wheat harvest (in the middle of July) and plowed in situ into the topsoil as a green manure by the middle of October that year. Inorganic fertilizers were annually and thoroughly incorporated into the soil before sowing spring wheat. The wheat–maize cropping pattern is described in detail in Supporting Information: Figure S2, and the contents of nutrients in each material is shown in Supporting Information: Table S1.

At the maturity of spring wheat in 2020, six random soil cores (5.0 cm in diameter) avoiding large roots were taken at 0–20 cm depth and mixed thoroughly as a composite sample. Fifteen soil samples were subsequently placed on ice to be transported to the lab and imminently sieved through a 2‐mm mesh to filter out impurities such as plant residues and rocks. Then, the soil was divided into three portions: 20 g of subsample was stored in a freezer at −80°C for DNA extraction, 80 g of fresh subsample was utilized to determine mineral N concentration and BNF rates, and the remaining sample was air‐dried and stored at room temperature for physicochemical analyses.

### Soil physicochemical analysis

Soil samples (about 0.50 g) were acidified with 1.0 mol L^−1^ HCl (20 ml) to remove carbonates and then were washed three to four times with distilled water till pH increased to a neutral reaction. SOC and TN were determined using the Elementar Analysensysteme (GmbH VarioEL). TP was extracted by mixed acid digestion (HF‐HClO_4_) and detected via the molybdenum blue colorimetry method [64]. Mineral N, including ammonium N and nitrate N, was extracted with 2 mol L^−1^ KCl and detected using a continuous flow analyzer (AA3; SEAL) [65]. The available phosphorus (AP) was extracted by 0.5 mol L^−1^ NaHCO_3_ and then detected via the molybdenum blue colorimetry method [66]. Soil pH was determined by a pH meter (LE438; Mettler‐Toledo Instruments) at a soil‐to‐water ratio of 1:2.5 (weight/volume) [65]. The treatment effects of soil properties are mentioned in Table 1.

**Table 1:** Physicochemical soil properties in different fertilization treatments

	CK	CF	CM	WS	GM
SOC (g kg^−1^)	12.20 (0.3)^c^	13.40 (1.80)^bc^	22.10 (2.20)^a^	15.70 (0.60)^b^	15.70 (0.20)^b^
TN (g kg^−1^)	1.38 (0.03)^c^	1.55 (0.17)^bc^	2.18 (0.32)^a^	1.73 (0.06)^b^	1.85 (0.05)^b^
TP (g kg^−1^)	1.35 (0.03)^b^	1.95 (0.09)^a^	2.58 (0.44)^a^	2.27 (0.11)^a^	2.04 (0.33)^a^
NH_4_^+^‐N (mg kg^−1^)	6.51 (1.30)^c^	17.10 (2.90)^b^	31.40 (0.90)^a^	9.04 (2.30)^c^	7.91 (3.10)^c^
NO_3_^−^‐N (mg kg^−1^)	19.70 (4.40)^b^	19.20 (1.90)^b^	34.9 (5.20)^a^	27.5 (4.20)^ab^	32.30 (2.80)^a^
AP (mg kg^−1^)	5.53 (0.30)^d^	27.60 (7.40)^c^	173.20 (5.20)^a^	67.80 (6.20)^b^	68.70 (4.50)^b^
pH	8.52 (0.01)^a^	8.44 (0.02)^b^	8.35 (0.04)^c^	8.40 (0.05)^bc^	8.41 (0.04)^bc^

*Note*: Data are mean ± SD in parentheses and different lowercases in a row indicate significant differences (least significant difference test, *p* < 0.05).

Abbreviations: CK, nonfertilization; CM, cow manure; Fertilization treatments—CF, chemical fertilizer; GM, green manure; WS, wheat straw; SOC, soil organic carbon; Soil properties—AP, available phosphorus; TN, total nitrogen, TP, total phosphorus.

### Determination of soil biological nitrogen fixation rates

We determined soil BNF rates based on the net changes in ^15^N of soil incubated with ^15^N_2_ gas [9]. Briefly, 2 g of soil was weighed and placed in a 12‐ml glass vial. Vials were then capped using rubber septum and septa screwed rings, vacuumized to remove ambient atmosphere, and immediately received synthetic gas comprising 20% (v/v) O_2_ and 80% (v/v) ^15^N_2_ (99 atom% ^15^N), while the reference vials received unlabeled N_2_, and all vials were maintained to a final pressure of 1 atm. Soil samples were incubated in the dark at room temperature for 22 days. Subsequently, vials were uncapped, and the soil samples were freeze‐dried and ground into a fine powder. The atom% ^15^N of the soil samples was analyzed using an elemental analyzer‐stable isotope mass spectrometer (Vario Isotope Cube‐Isoprime, Elementar). The BNF rates were calculated as follows:

$$\text{BNF}\;\text{rate}\left(\mathrm{\mu g}\;N\;{\text{kg}}^{-1}\text{soil}\;d^{-1}\right)\;={(}^{15}N\;\mathrm{atom}\%\mathrm{Soil}{–}^{15}N\;\mathrm{atom}\%{\mathrm{Soil}}_{\mathrm{control}})\times \mathrm{TN})/T,$$

where ^15^N atom% Soil and ^15^N atom% Soil_control_ are the ^15^N atom excess (%) of the ^15^N_2_ labeled and unlabeled soil samples, respectively; TN is the soil total N content after incubation, and *T* is the incubation time.

### DNA extraction and real‐time fluorescent quantitative polymerase chain reaction (qPCR)

Soil microbial DNA was extracted from 0.5 g of soil using FastDNA Spin Kit (MP Bio) following the manufacturer's instructions. The *nifH* (for diazotrophs) and *18S rRNA* (for AMF) genes were analyzed by real‐time quantitative polymerase chain reaction (qPCR) on a Line‐Gene 9600 Plus Real‐time PCR system (Bioer), using primer pairs *nifH*‐F/*nifH*‐R (5′‐AAAGGYGGWATCGGYAARTCCACCAC‐3′/5′‐TTGTTSGCSGCRTACATSGCCATCAT‐3′) [67] and *AMV4.5N*‐F/*AMDG*‐R (5′‐AAGCTCGTAGTTGAATTTCG‐3′/5′‐CCCAACTATCCCTATTAATCAT‐3′) [68] to quantify the diazotrophs and arbuscular mycorrhizal fungi abundance as previous study [10, 69], respectively. The qPCR reaction system contained 5 μl of ChamQ SYBR qPCR Master Mix (Vazyme Biotech Co., Ltd.), 1 μl of template DNA, 0.2 μl of primer F (10 μM), 0.2 μl of primer R (10 μM), and 3.6 μl of double distilled H_2_O (ddH_2_O). The amplification of the *nifH* gene fragment was performed at 95°C for 5 min, followed by 40 cycles of denaturation at 95°C for 15 s, annealing at 60°C for 30 s, and extension at 72°C for 40 s. The amplification of the *18S rRNA* gene was performed at 95°C for 5 min, followed by 40 cycles of 95°C for 10 s, 60°C for 30 s, and 72°C for 40 s. A 10× dilution series of a recombinant plasmid carrying the *nifH* or *18S rRNA* gene was amplified to create a standard curve. Standard curves showed an amplification efficiency of 96.4% (*R* ^2^ = 0.99) for the *nifH* gene, and 92.5% (*R* ^2^ = 0.99) for the *18S rRNA* gene, respectively.

### Illumina MiSeq sequencing and bioinformatics analyses

Consistent with qPCR, the *nifH* and *18S rRNA* genes were amplified using the same primer pairs *nifH‐*F/*nifH‐*R and *AMV4.5N*‐F/*AMDG*‐R, respectively. PCR reactions were carried out in 25‐μl reaction volumes consisting of 12.5 μl of the KAPA2G Robust HotStart ReadyMix PCR (KAPA biosystems), 1 μl each of 5 μM forward and reverse primers, 5 μl of template DNA (30 ng), and 5.5 μl of ddH_2_O. The cycling parameters were 95°C for 3 min, followed by 35 cycles of 95°C for 30 s, 55°C for 30 s, 72°C for 45 s, and a final extension at 72°C for 10 min. The PCR amplifications were carried out in triplicates, and the amplicons were purified and pooled. Sequencing libraries were generated using NEB Next Ultra II DNA Library Prep Kit (New England Biolabs, Inc.) following the manufacturer's recommendations. The library quality was assessed by Nanodrop 2000 (ThermoFisher Scientific, Inc.), Agilent 2100 Bioanalyzer (Agilent Technologies, Inc.), and the ABI StepOnePlus Real‐Time PCR System (Applied Biosystems, Inc.), successively. The library was finally paired‐end sequenced using an Illumina MiSeq PE300 platform (Allwegene Company in Beijing). Raw sequencing data have been submitted to the NCBI Sequence Read Archive (Study ID: SRP391822 for diazotrophs and SRP391816 for AMF).

After sequencing, nucleotide sequences were imported into QIIME2 [70], demultiplexed, and denoised using DADA2 [71]. High‐quality sequences were acquired by filtering out the low‐quality sequences (short sequences <200 bp length, reads with an average quality score <20, or containing ambiguous nucleotides) and chimeric sequences. Following quality control, in total, 885,340 and 1,952,575 high‐quality sequences were received from all diazotrophic and AMF samples, respectively (Supporting Information: Table S2). Then, the number of sequences were standardized across samples to account for different sequencing depth by randomly subsampling each sample to the lowest number of sequences counts obtained by any sample. The valid sequences were clustered into amplicon sequence variants (ASVs) with a sequence identity threshold of 100% via DADA2 [36, 71]. Across all soil samples, we obtained 526 and 411 ASVs in total for diazotrophs and AMF, respectively. The corresponding representative sequences and ASVs table were generated, and ASVs tables were subsampled by rarefaction analyses (Supporting Information: Figure S3). Taxonomy was assigned by the Ribosomal Database Project naïve Bayesian classifier referring to the GeneBank Database (http://fungene.cme.msu.edu/) for *nifH* gene libraries [72] and the MaarjAM Glomeromycota DNA sequence Database (http://maarjam.botany.ut.ee) for *18S rRNA* libraries [73]. Taxonomic analysis revealed that about 96% of the *nifH* gene reads and 95% of the *18S rRNA* gene reads could be classified into six bacterial phyla (Proteobacteria, Actinobacteria, Firmicutes, Chlorobi, Cyanobacteria, and Verrucomicrobia) and two fungal classes (Glomeromycetes and Basidiobolomycetes), respectively.

### Co‐occurrence network analysis

A co‐occurrence network with all the samples was constructed via the “WGCNA” package in R. We focused on microbial phylotypes (ASVs) with more than 0.01% relative abundances of the total number for *nifH* or *18S rRNA*. The diazotrophic and AMF ASVs were combined into a new abundance table to finally contain 307 diazotrophic ASVs and 234 AMF ASVs. Then all pair‐wise correlations among ASVs were calculated based on Spearman's method, and *P*‐values were adjusted by Benjamini and Hochberg's false discovery rate (FDR) for multiple testing [74]. We set the cutoff for *r*‐values (Spearman's coefficient) and adjusted *p*‐values to 0.65 and 0.01, respectively. We chose that the cutoff, which has been widely used in literature and is comparable across studies [9], would have both mathematical and biological meanings and reveal the microbial phylotypes (ASVs) that strongly co‐occur and are more likely to interact with each other. The “Gephi” software (https://gephi.org/) was employed to identify and visualize the main ecological clusters (modules) in the co‐occurrence network. The relative abundances (*z*‐score standardization, *x*′ = (*x* − average)/SD)) of the ASVs were averaged within each ecological cluster to represent the relative abundance of each module. When an ecological cluster positively correlated well with soil BNF rates, we designated all species within the ecological cluster as keystone phylotypes and regarded the cluster as the key ecological cluster [33, 35].

### Statistical analyses

Mean values for each variable, including soil property and the abundance of functional genes were compared by Fisher's LSD test (*p* < 0.05) using SAS (version 8.1). To test for differences in alpha diversity between fertilizations, we used the Wilcoxon rank‐sum test in R. The STAMP (v. 2.1.3) was adopted for determining statistical differences in the relative abundance of microbes after long‐term fertilization. All pair‐wise Spearman's correlations among soil property, alpha diversity, the relative abundance of the module, and the BNF rate were calculated using Origin 2022. PERMANOVA based on Bray‐Curtis distance and Mantel test were performed using the “vegan” package in R. Random Forest analysis was performed to identify the optimal set of ASVs correlated to the BNF rate using the “randomForest” package in R. The percentage increases in the mean squared error (%IncMSE) were used to rank the importance of these ASVs, that is, higher %IncMSE implies more important ASVs, and the significance of each predictor (ASV) on the response variables (BNF rate) was assessed with 5000 trees [75]. Phylogenetic sampling theory was analytically performed to evaluate the degree to which AMF and diazotrophic communities presented clustering (<−2), randomness (>−2 and <2), or over‐dispersion (>2) by using the “picante” package in R [76]. Then the differences between observed and expected phylogenetic diversity (PD) for each module were calculated and compared. When the observed PD is greater than the expected PD (i.e., >2), the microbial community in the module is deemed to be phylogenetically over‐dispersed, implying competitive exclusion for closely related taxa [52].

The PLS‐PM analysis was performed by the “plspm” package in R [77] to assess the direct and indirect effects of soil property, microbial community, and relative abundance of diazotrophs and AMF within the main module, and their synergistic function on soil BNF rate. The overall model goodness‐of‐fit (GOF) index and *R* ^2^ coefficient of PLS‐PM were evaluated, and GOF > 0.7 was considered the acceptable value for the PLS‐PM [77]. In addition, the indirect and cross‐loading effects of soil properties, functional microbial communities, and relative abundance of diazotrophs and AMF within the main module, and their synergistic function on soil BNF rate were also calculated to further explain the PLS‐PM using the “plspm” package [78].

## AUTHOR CONTRIBUTIONS

Guopeng Zhou and Weidong Cao designed the research. Guopeng Zhou, Songjuan Gao, Danna Chang, Ting Liang, Shun Li, Jiudong Zhang, Zongxian Che, and Weidong Cao performed the research. Guopeng Zhou, Kunkun Fan, Guilong Li, and Hai Liang conducted data analysis. Guopeng Zhou, Kunkun Fan, and Guilong Li wrote the manuscript and Weidong Cao edited the manuscript. All authors have commented on and approved the final manuscript.

## CONFLICT OF INTEREST

The authors declare no conflict of interest.

## Supporting information

Supporting information.

Supporting information.

## Data Availability

The raw sequences were archived in NCBI Sequence Read Archive (SRA) with accession numbers SRP391822 and SRP391816 (https://www.ncbi.nlm.nih.gov/sra/?term=SRP391822 and https://www.ncbi.nlm.nih.gov/sra/?term=SRP391816). Supporting Information (figures, tables, graphical abstract, videos) may be found in the online DOI or iMeta Science http://www.imeta.science/.
