# Supplementary material for: Synergistic effects of diazotrophs and arbuscular mycorrhizal fungi on soil biological nitrogen fixation after three decades of fertilization
Source: Imeta. 2023 Jan 27;2(1):e81. doi: 10.1002/imt2.81 (PMC10989903; doi:10.1002/imt2.81)
Supplement: Supplementary file 2 — Supporting information. [file IMT2-2-e81-s001.docx]

**Title**

Synergistic effects of diazotrophs and arbuscular mycorrhizal fungi on soil biological nitrogen fixation after three decades of fertilization

**Running title**

Synergistic effects of diazotrophs and AMF on soil nitrogen fixation

**Authors**

Guopeng Zhou^1^, Kunkun Fan^2^, Guilong Li^3^, Songjuan Gao^4^, Danna Chang^1^, Ting Liang^1^, Shun Li^4^, Hai Liang^4^, Jiudong Zhang^5^, Zongxian Che^5^, Weidong Cao^1*^

**Affiliations**

^1^ Key Laboratory of Plant Nutrition and Fertilizer, Ministry of Agriculture and Rural Affairs / Institute of Agricultural Resources and Regional Planning, Chinese Academy of Agricultural Sciences, Beijing, 100081, China.

^2^ State Key Laboratory of Soil and Sustainable Agriculture, Institute of Soil Science, Chinese Academy of Sciences, Nanjing 210008, China.

^3^ Institute of Soil&Fertilizer and Resource&Environment, Jiangxi Academy of Agricultural Sciences, Nanchang 330200, China.

^4^ College of Resources and Environmental Sciences, Nanjing Agricultural University, Nanjing, 210095, China.

^5^ Institute of Soil and Fertilizer and Water-saving Agriculture, Gansu Academy of Agriculture Science, Lanzhou, 730070, PR China.

****Corresponding author***

Weidong Cao (caoweidong@caas.cn; Tel: +86 10 82109622; Fax: +86 10 82106733)

Table S1 The average contents of nutrients in each material (dry basis).

|  | **Nitrogen content (g kg^-1^)** | **Phosphorous content (g kg^-1^)** | **Potassium content (g kg^-1^)** | **Moisture content (%)** |
| --- | --- | --- | --- | --- |
| **Cow manure** | 14.8 | 3.22 | 23.0 | 20.1 |
| **Wheat straw** | 5.32 | 2.24 | 12.7 | 10.0 |
| **Green manure** | 32.9 | 2.60 | 36.1 | 83.0 |

Table S2 The number of high-quality sequences and ASVs received from each diazotrophic and AMF sample. Fertilization treatments: CK, non-fertilization; CF, chemical fertilizer; CM, cow manure; WS, wheat straw; GM, green manure.

| **Sample ID** | **High-quality sequences** | | **ASVs** | |
| --- | --- | --- | --- | --- |
|  | **Diazotrophs** | **AMF** | **Diazotrophs** | **AMF** |
| **CK1** | 27442 | 80160 | 206 | 251 |
| **CK2** | 26458 | 88384 | 192 | 232 |
| **CK3** | 55026 | 74320 | 235 | 264 |
| **CF1** | 86891 | 88652 | 195 | 197 |
| **CF2** | 84212 | 159298 | 232 | 185 |
| **CF3** | 72986 | 80964 | 234 | 158 |
| **CM1** | 23621 | 115073 | 297 | 173 |
| **CM2** | 23656 | 81788 | 288 | 200 |
| **CM3** | 25508 | 122624 | 277 | 167 |
| **WS1** | 52072 | 235955 | 254 | 184 |
| **WS2** | 94930 | 193265 | 280 | 183 |
| **WS3** | 31091 | 220678 | 284 | 163 |
| **GM1** | 87603 | 75126 | 254 | 242 |
| **GM2** | 94881 | 196317 | 259 | 164 |
| **GM3** | 98963 | 139971 | 279 | 199 |

Table S3 The differences of community structures of diazotrophs and arbuscular mycorrhizal fungi among fertilization treatments assessed by permutational multivariate analysis of variance (PERMANOVA). Fertilization treatments: CK, non-fertilization; CF, chemical fertilizer; CM, cow manure; WS, wheat straw; GM, green manure.

|  | **Diazotrophs** | | **Arbuscular mycorrhizal fungi** | |
| --- | --- | --- | --- | --- |
|  | **R statistic** | **P value** | **R statistic** | **P value** |
| **all** | 0.524 | **0.001** | 0.315 | **0.001** |
| **CK vs. CF** | 0.135 | 0.253 | 0.927 | **0.028** |
| **CK vs. CM** | 0.990 | **0.032** | 0.594 | **0.033** |
| **CK vs. WS** | 0.375 | 0.060 | 0.479 | **0.026** |
| **CK vs. GM** | 0.615 | **0.025** | 0.667 | **0.028** |
| **CF vs. CM** | 0.979 | **0.024** | 0.010 | 0.562 |
| **CF vs. WS** | 0.240 | 0.137 | 0.229 | 0.121 |
| **CF vs. GM** | 0.438 | 0.065 | -0.104 | 0.802 |
| **CM vs. WS** | 0.724 | **0.021** | -0.063 | 0.661 |
| **CM vs. GM** | 0.875 | **0.024** | 0.083 | 0.246 |
| **WS vs. GM** | 0.125 | 0.247 | 0.031 | 0.360 |

Table S4 Taxonomic α-diversity indices of diazotrophic communities and arbuscular mycorrhizal fungal communities after thirty-three years of fertilization. Fertilization treatments: CK, non-fertilization; CF, chemical fertilizer; CM, cow manure; WS, wheat straw; GM, green manure.

| Taxa | α diversity index | Treatment1 | Treatment1_mean | Treatment2 | Treatment2_mean | P value |
| --- | --- | --- | --- | --- | --- | --- |
| Diazotrophs | Chao1 | CK | 231.7 | CF | 263.2 | 0.47668 |
| Diazotrophs | Chao1 | CK | 231.7 | CM | 317.8 | **0.01413** |
| Diazotrophs | Chao1 | CK | 231.7 | WS | 310.9 | **0.02333** |
| Diazotrophs | Chao1 | CK | 231.7 | GM | 309.2 | **0.04139** |
| Diazotrophs | Observed_species | CK | 211 | CF | 220 | 0.62998 |
| Diazotrophs | Observed_species | CK | 211 | CM | 287 | **0.00272** |
| Diazotrophs | Observed_species | CK | 211 | WS | 273 | **0.00519** |
| Diazotrophs | Observed_species | CK | 211 | GM | 264 | **0.01249** |
| Diazotrophs | Shannon | CK | 5.36 | CF | 5.67 | 0.36614 |
| Diazotrophs | Shannon | CK | 5.36 | CM | 6.34 | **0.03323** |
| Diazotrophs | Shannon | CK | 5.36 | WS | 6.29 | 0.09609 |
| Diazotrophs | Shannon | CK | 5.36 | GM | 6.17 | **0.04729** |
| Diazotrophs | Simpson | CK | 0.95 | CF | 0.96 | 0.41072 |
| Diazotrophs | Simpson | CK | 0.95 | CM | 0.97 | 0.12434 |
| Diazotrophs | Simpson | CK | 0.95 | WS | 0.98 | 0.09445 |
| Diazotrophs | Simpson | CK | 0.95 | GM | 0.97 | 0.12434 |
| AMF | Chao1 | CK | 274.9 | CF | 210.6 | **0.00728** |
| AMF | Chao1 | CK | 274.9 | CM | 202.1 | **0.00815** |
| AMF | Chao1 | CK | 274.9 | WS | 206.6 | **0.00206** |
| AMF | Chao1 | CK | 274.9 | GM | 236.7 | 0.30529 |
| AMF | Observed_species | CK | 249 | CF | 180 | **0.00960** |
| AMF | Observed_species | CK | 249 | CM | 180 | **0.00741** |
| AMF | Observed_species | CK | 249 | WS | 177 | **0.00330** |
| AMF | Observed_species | CK | 249 | GM | 202 | 0.12434 |
| AMF | Shannon | CK | 5.28 | CF | 4.53 | **0.01363** |
| AMF | Shannon | CK | 5.28 | CM | 4.82 | 0.07998 |
| AMF | Shannon | CK | 5.28 | WS | 4.25 | **0.00831** |
| AMF | Shannon | CK | 5.28 | GM | 4.90 | **0.01089** |
| AMF | Simpson | CK | 0.96 | CF | 0.92 | **0.01417** |
| AMF | Simpson | CK | 0.96 | CM | 0.94 | 0.14470 |
| AMF | Simpson | CK | 0.96 | WS | 0.90 | 0.09445 |
| AMF | Simpson | CK | 0.96 | GM | 0.95 | 0.37390 |

Table S5 The co-occurrence network properties for the main ecological clusters of the diazotrophic and arbuscular mycorrhizal fungal communities.

|  | **Nodes** | **Edges** | **Density** | | **Positive link** | **Negative link** |
| --- | --- | --- | --- | --- | --- | --- |
| **Module #0** | 118 | 1064 | 0.154 | | 1063 | 1 |
| **Module #1** | 97 | 512 | 0.110 | | 511 | 1 |
| **Module #2** | 169 | 960 | 0.068 | | 925 | 35 |
| **Module #3** | 74 | 439 | 0.163 | | 439 | 0 |
| **Links between the main ecological clusters** | | | | **Total links** | **Positive links** | **Negative links** |
| **Module #0 to Module #1** | | | | 136 | 108 | 28 |
| **Module #0 to Module #2** | | | | 620 | 8 | 612 |
| **Module #0 to Module #3** | | | | 34 | 13 | 21 |
| **Module #1 to Module #2** | | | | 302 | 40 | 262 |
| **Module #1 to Module #3** | | | | 24 | 13 | 11 |
| **Module #2 to Module #3** | | | | 368 | 21 | 347 |

Table S6 The statistics of positive links between diazotrophs and arbuscular mycorrhizal fungi in/among main ecological clusters.

|  | **Positive links between diazotrophic and arbuscular mycorrhizal fungal ASVs** | **Node (Diazotrophs)** | **Node (AMF)** |
| --- | --- | --- | --- |
| **Total** | 293 | 152 | 137 |
| **In Module #0** | 15 | 26 | 9 |
| **In Module #1** | 60 | 49 | 13 |
| **In Module #2** | 88 | 42 | 69 |
| **In Module #3** | 31 | 15 | 30 |
| **Module #0 to Module #1** | 2 | / | / |
| **Module #0 to Module #2** | 3 | / | / |
| **Module #0 to Module #3** | 9 | / | / |
| **Module #1 to Module #2** | 21 | / | / |
| **Module #1 to Module #2** | 8 | / | / |
| **Module #2 to Module #3** | 5 | / | / |
|  | **Negative links between diazotrophic and arbuscular mycorrhizal fungal ASVs** | **Node (Diazotrophs)** | **Node (AMF)** |
| **Total** | 266 | 134 | 119 |

Table S7 The list of amplicon sequence variants (ASVs) with positive relationships between diazotrophs and AMF in co-occurrence pattern of diazotrophic–arbuscular mycorrhizal fungal network.

Please see the excel file (SI_Table S7.xlsx).

Table S8 The relative abundance of ASVs with positive relationships between diazotrophs and AMF in co-occurrence pattern of diazotrophic–arbuscular mycorrhizal fungal network. Data are means ± SD in parentheses and different lowercases in a row indicate significant differences (least significant difference test, *P* < 0.05). Fertilization treatments: CK, non-fertilization; CF, chemical fertilizer; CM, cow manure; WS, wheat straw; GM, green manure.

|  | **CK** | **CF** | **CM** | **WS** | **GM** |
| --- | --- | --- | --- | --- | --- |
| **Diazotrophs** (%) | | | | | |
| **Module #0** | 2.55 (0.77)c | 4.43 (2.21)bc | 5.87 (1.38)b | 5.83 (1.85)b | 7.19 (1.23)a |
| **Module #1** | 3.03 (0.85)c | 6.29 (6.41)bc | 16.99 (3.84)a | 11.83 (7.06)ab | 3.30 (0.39)c |
| **Module #2** | 43.3 (7.95)a | 31.94 (2.69)b | 4.92 (2.75)d | 16.95 (4.73)c | 14.44 (6.70)cd |
| **Module #3** | 1.35 (1.08)c | 5.84 (1.29)a | 2.34 (1.31)bc | 4.80 (0.54)ab | 4.02 (2.47)abc |
| **Other** | 1.18 (0.65)a | 1.14 (0.64)a | 0.85 (0.49)a | 0.65 (0.10)a | 1.03 (0.21)a |
| **Total** | 51.41 (4.69)a | 49.64 (6.83)a | 30.97 (6.92)b | 40.05 (6.5)ab | 29.98 (7.68)b |
| **Arbuscular mycorrhizal fungi** (%) | | | | | |
| **Module #0** | 1.71 (0.37)b | 11.43 (3.93)ab | 7.25 (4.23)ab | 16.50 (14.19)a | 9.54 (3.96)ab |
| **Module #1** | 1.76 (1.53)a | 0.65 (0.87)a | 5.48 (2.71)a | 2.99 (2.88)a | 2.89 (4.47)a |
| **Module #2** | 51.78 (5.08)a | 27.36 (13.46)b | 22.91 (20.84)b | 32.51 (7.12)b | 33.56 (4.76)b |
| **Module #3** | 2.60 (2.09)b | 19.50 (4.19)ab | 27.49 (16.21)a | 18.11 (9.08)ab | 15.59 (8.20)ab |
| **Other** | 1.22 (1.53)a | 0.66 (0.59)a | 6.35 (8.17)a | 0.96 (0.91)a | 1.07 (1.52)a |
| **Total** | 59.07 (1.69)a | 59.6 (15.98)a | 69.48 (3.88)a | 71.06 (11.15)a | 62.65 (9.54)a |

Table S9 Amplicon sequence variant (ASV) abundance properties of significant diazotrophic species for nitrogen fixation rates based on the Random Forest model in the keystone ecological clusters (Modules #0 and #3).

Please see the excel file (SI_Table S9.xlsx).

Table S10 Amplicon sequence variant (ASV) abundance table of diazotrophic and AMF species in the main ecological clusters.

Please see the excel file (SI_Table S10.xlsx).

**Figure S1 Seasonal variation in the daily precipitation and temperature during the growing season (from March to October) of 2020.**

**Figure S2 The cropping pattern of the long-term fertilization treatment.** Apart from green manure (hairy vetch) treatment, there is fallow after spring wheat harvest in other treatments. Fertilization treatments: CK, non-fertilization; CF, chemical fertilizer; CM, cow manure; WS, wheats straw; GM, green manure.

**Figure S3 Rarefaction curves showing the observed number of ASVs in different samples.** Fertilization treatments: CK, non-fertilization; CF, chemical fertilizer; CM, cow manure; WS, wheats straw; GM, green manure.

**Figure S4 Statistically significant differences in the relative abundance of Diazotrophs and AMF at genus levels after long-term fertilizations.** Fertilization treatments: CK, non-fertilization; CF, chemical fertilizer; CM, cow manure; WS, wheats straw; GM, green manure.

**Figure S5 Pairwise comparisons of soil properties are shown, with a color gradient denoting Spearman’s correlation coefficients.** (A) Taxonomic community compositions of diazotrophs and arbuscular mycorrhizal fungi were related to each soil property by Mantel tests. (B) Taxonomic community compositions of the main ecological clusters were related to each soil property by Mantel tests. Edge width corresponds to the Mantel’s r statistic for the corresponding distance correlations, and edge color denotes the statistical significance based on 9,999 permutations. AMF: arbuscular mycorrhizal fungi. Soil properties: SOC, soil organic carbon; TN, total nitrogen, TP, total phosphorus; AP, available phosphorus; AN, ammonium nitrogen; NN, nitrite nitrogen; N/P, the nitrogen to phosphorus rate.

**Figure S6 Co-occurrence network interactions of soil diazotrophs and arbuscular mycorrhizal fungi.** (A) Co-occurrence patterns in soil diazotrophs and arbuscular mycorrhizal fungi. (B) The sub-network size (numbers of node) and connectivity (numbers of edge) within the co-occurrence network.

A connection stands for a strong correlation coefficient (spearman’s r) greater than 0.65 or less than -0.65 and a *P* value of less 0.01. The co-occurrence networks are colored by main module. The nodes represent unique ASVs in the data sets. The size of each node is proportional to the relative abundance. Bars with different letters reveal the values that differ significantly among fertilization treatments at *P* < 0.05 (LSD test).

**Figure S7 Relative abundance of the diazotrophs and arbuscular mycorrhizal fungi in the main ecological clusters in different fertilization treatments.** From left to right are Modules #0, #1, #2, and #3, represented by red, blue, green, and yellow plots, respectively. Different lowercases indicate the values that differ significantly among treatments at *P* < 0.05 (least significant difference test). AMF: arbuscular mycorrhizal fungi. Fertilization treatments: CK, non-fertilization; CF, chemical fertilizer; CM, cow manure; WS, wheats straw; GM, green manure.

**Figure S8 Mantel tests describing the soil properties in affecting taxonomic community compositions.** (A) Taxonomic community compositions of the diazotrophs and arbuscular mycorrhizal fungi (with positive correlation between them) were related to each soil property by Mantel tests. (B) Taxonomic community compositions of the diazotrophs with positive links to AMF in four ecological clusters were related to each soil property by Mantel tests. (C) Taxonomic community compositions of the arbuscular mycorrhizal fungi with positive links to diazotrophs in four ecological clusters were related to each soil property by Mantel tests. Edge width corresponds to the Mantel’s r statistic for the corresponding distance correlations, and edge color denotes the statistical significance based on 9,999 permutations. AMF: arbuscular mycorrhizal fungi. Soil properties: SOC, soil organic carbon; TN, total nitrogen, TP, total phosphorus; AP, available phosphorus; AN, ammonium nitrogen; NN, nitrite nitrogen; N/P, the nitrogen to phosphorus rate.

**Figure S9 Spearman correlation between physicochemical soil properties, relative abundance of the main ecological clusters, and N fixation rates.** Significance level of predictor is **P* < 0.05. nifh, diazotrophs; AMF, arbuscular mycorrhizal fungi; Posi, positive (diazotrophs with positive links to AMF or AMF with positive links to diazotrophs). Soil properties: SOC, soil organic carbon; TN, total nitrogen, TP, total phosphorus; AP, available phosphorus; AN, ammonium nitrogen; NN, nitrite nitrogen; N/P, the nitrogen to phosphorus rate.

**Figure S10 Spearman correlation between physicochemical soil properties, diazotrophs and arbuscular mycorrhizal fungal alpha-diversity, and N fixation rates.** Significance level of predictor is **P* < 0.05. nifh, diazotrophs; AMF, arbuscular mycorrhizal fungi. Soil properties: SOC, soil organic carbon; TN, total nitrogen, TP, total phosphorus; AP, available phosphorus; AN, ammonium nitrogen; NN, nitrite nitrogen; N/P, the nitrogen to phosphorus rate.
